# Development and Regulation of Novel Influenza Virus Vaccines: A United States Young Scientist Perspective

**DOI:** 10.3390/vaccines6020024

**Published:** 2018-04-27

**Authors:** Surender Khurana

**Affiliations:** Division of Viral Products, Center for Biologics Evaluation and Research (CBER), Food and Drug Administration (FDA), Silver Spring, MD 20993, USA; Surender.Khurana@fda.hhs.gov

**Keywords:** influenza, vaccine, correlates, universal, next-generation, animal model, human challenge, adjuvants, vaccine safety, regulatory pathway

## Abstract

Vaccination against influenza is the most effective approach for reducing influenza morbidity and mortality. However, influenza vaccines are unique among all licensed vaccines as they are updated and administered annually to antigenically match the vaccine strains and currently circulating influenza strains. Vaccine efficacy of each selected influenza virus vaccine varies depending on the antigenic match between circulating strains and vaccine strains, as well as the age and health status of the vaccine recipient. Low vaccine effectiveness of seasonal influenza vaccines in recent years provides an impetus to improve current seasonal influenza vaccines, and for development of next-generation influenza vaccines that can provide broader, long-lasting protection against both matching and antigenically diverse influenza strains. This review discusses a perspective on some of the issues and formidable challenges facing the development and regulation of the next-generation influenza vaccines.

## 1. Introduction

The Food and Drug Administration (FDA) is responsible for regulating vaccines in the United States. In 1945, the first inactivated influenza vaccine (IIV) was licensed in the United States (US). A live attenuated influenza vaccine (LAIV) was licensed in 2003. Since 2010, US Centers for Disease Control and Prevention (CDC) has recommended annual immunization with current licensed influenza vaccines for all individuals 6 month and older. The seasonal influenza vaccines licensed in the US contain two Influenza A strains (one H1 and one H3) and either one or two Influenza B antigens in trivalent or quadrivalent formulations, respectively. The current inactivated influenza vaccines are licensed and released based on hemagglutinin (HA) content alone since HA is considered the key protective antigen for the current inactivated influenza vaccines. Conventional influenza vaccine production relies on an egg-based manufacturing process that yields inconsistent amounts of hemagglutinin (HA). The adaptation of vaccine strains to growth in eggs to increase HA yields, can result in accumulation of mutations that could impact important antigenic sites and may reduce the protective immunity against the circulating influenza strains. In addition, there is a significant time lag between virus strain selection by the WHO and FDA Vaccine Advisory Committee (VRBPAC) and the availability of vaccine for mass vaccination. The current production of egg-derived influenza virus vaccines is a slow, inefficient process and has the potential of bottlenecks at various steps during the production process and lot release. To overcome some of these limitations, cell-based and recombinant production technologies were developed for more rapid production of seasonal influenza vaccines that could also be used in response to a pandemic threat. The first influenza vaccine produced in mammalian cell culture received licensure in November 2012 in the United States. The first recombinant influenza vaccine based exclusively on hemagglutinin protein was licensed in 2013 by FDA. To improve vaccine immunogenicity and protection in the elderly population, the first adjuvanted seasonal influenza vaccine combined with adjuvant, MF59C.1., an oil in water emulsion of squalene, was licensed in November 2015 by FDA for adults 65 years of age and older.

## 2. Vaccine Antigens

Hemagglutinin (HA) and Neuraminidase (NA) surface glycoproteins of influenza virus evolve and re-assort, leading to antigenic drift and antigenic shift. For the currently licensed influenza vaccine to be effective in protection, the vaccine’s HA and NA antigens needs to be derived from a strain that is well-matched to a circulating influenza strain. This will ensure that the antibodies elicited will protect against infection from that strain. Because multiple influenza strains are in constant circulation, seasonal influenza vaccine is produced and administered annually to protect against the three/four influenza strains expected to be most prevalent that year (i.e., a trivalent/quadrivalent influenza vaccine). Several seasonal quadrivalent influenza vaccine (containing one H1, one H3, and two B influenza antigens) and trivalent influenza vaccine (containing one H1, one H3, and one B influenza antigen) formulations are licensed for immunizations of different age groups in the US (Table 1 and Table 2, respectively). Every year, the vaccine manufacturers submit a supplement under their licensure to update the influenza virus strains to be included in their vaccines. This strain change supplement must be approved by FDA before the influenza vaccine containing new virus antigens can be released to market. No additional clinical data specific for the new strains is required for inactivated and recombinant protein-based seasonal influenza vaccines. However, a small clinical study in adults is required to verify adequate attenuation of licensed LAIV strains.

The process of annual seasonal influenza vaccine production and regulation is very intense and time consuming with limited flexibility [1]. The difficulty in predicting prevalent circulating strains has resulted in mismatch between the annual vaccine virus strains and circulating viruses resulting in a vaccine with variable efficacy and durability, requiring annual reformulation and boosting [2]. There is widespread recognition that a more broadly protective influenza vaccine that does not require annual selection and reformulation would be a valuable contribution to public health [3,4].

Current vaccine strategies are based on inducing hemagglutination-inhibiting (HAI) antibodies as the primary immunological endpoint. HAI antibody is a surrogate for blocking of influenza virus binding to sialic acid receptor on the surface of red blood cells. The selection of red blood cells used for the HAI assay may be critical for accurate measurements of antibodies blocking virus-receptor interactions due to differential preference of sialic acid receptors present on various type of red blood cells by diverse influenza strains. Other virus neutralization assays have been developed, but thus far have not been used as the primary immunological endpoint for influenza vaccine licensure.

In addition to the currently licensed seasonal influenza inactivated vaccines (split or subunit vaccines), several influenza vaccine candidates are currently in preclinical or early clinical development. Some target conserved influenza proteins, such as NP, M1, M2e as well as surface glycoproteins NA and conserved regions within hemagglutinin stem (HA2) and HA1 receptor binding domain with the hope of eliciting broadly cross-protective antibody and/or T cell responses [5,6,7]. Other new vaccine platforms include synthetic peptides, virus-like particles (VLPs), DNA vectors, messenger RNA, viral vectors, recombinant proteins, and live-attenuated or inactivated influenza viruses in presence or absence of adjuvants. A next-generation influenza vaccine that is safe and elicits long-lasting broad protective immune response against future drifted and pandemic strains of influenza in all age groups is highly desirable. An ideal vaccine should interact with more diverse T cells and/or antibodies and elicit long-lasting high affinity antibodies to protective targets within influenza. Additionally, desired properties of any new influenza virus vaccine include reduced antigen dose and the number of vaccinations that is amenable for mass vaccinations. This perspective describes some of the formidable challenges for the regulation of next-generation influenza vaccines under development.

## 3. Adjuvants

Adjuvants can act as immune potentiators and reduce the vaccine dosage needed to stimulate an immune response. Several adjuvants are being evaluated for the next-generation influenza vaccines [6,7]. Adjuvants are not considered as active ingredients based on the Code of Federal Regulations (CFR) Title 21 CFR 610.15. They are included as constituent material (preservatives, diluents, adjuvants). An adjuvant shall not be introduced into a product unless there is satisfactory evidence that it does not adversely affect the safety or potency of the product and evaluation of “added benefit” (justification for use of the adjuvant) should be demonstrated. It is the adjuvanted vaccine formulation, in toto, that is tested in clinical trials and licensed.

Manufacturers should provide a rationale for the use of adjuvant in their vaccine formulation, with supportive data that may be derived from preclinical studies (proof of concept studies in animal models). Mode of action (MOA) studies in vivo and in vitro are important, but not absolutely required prior to entering clinical trials. Safety and immunogenicity data from use of the same adjuvant with related vaccine antigens, if available, can be supportive. Early clinical immunogenicity trials should be performed to compare adjuvanted vs. unadjuvanted vaccines to provide evidence of enhanced immune response, antigen sparing effects, or other advantages. Since these studies are designed to provide safety information, data can be used to select the optimal dose of both antigen and adjuvant that are both immunogenic and tolerable (least frequency of serious adverse reactions in vaccine recipients). Because adjuvants are not considered active ingredients from a regulatory perspective, manufacturers are not required to demonstrate the “added benefit” of an adjuvant in comparative phase 3 efficacy trials, e.g., studies comparing vaccine antigen with and without adjuvant, but it is necessary for sponsors to demonstrate the added benefit of the adjuvant in early phase studies or in a well-defined animal model. 

Preclinical safety should be evaluated as per 21 CFR 312.23(a)(8) and conducted prior to clinical trials to identify and characterize potential local and systemic adverse effects (SAEs). These studies should include repeat dose toxicity studies and histopathology of full tissue list based on WHO guidance for novel adjuvants with special consideration of reproductive toxicity testing [8].

The clinical safety of the adjuvanted vaccine must be demonstrated in pre-licensure studies as per Safety requirement for vaccine licensure (21 CFR 600.3). There are special considerations for safety evaluation of adjuvanted vaccines including suggested comparisons between adjuvanted vaccine vs. unadjuvanted antigen vs. saline placebo, and specific clinical follow up regarding any identified symptoms consistent with autoimmune and neuroinflammatory diseases. A longer post-vaccination follow-up, typically 12 months following last vaccination is recommended to capture delayed vaccine associated serious adverse events (SAE) and new onset of medical conditions. However, some potential adverse events beginning after vaccination may not be recognized or diagnosed until much later. Adverse events of “special interest” (AESI) with focus on autoimmune/autoinflammatory diseases such as neuroinflammatory disorders (e.g., optic neuritis, transverse myelitis), musculoskeletal and connective tissue diseases (e.g., rheumatoid arthritis, SLE, Wegener’s), and gastrointestinal disorders (e.g., Crohn’s disease, ulcerative colitis) should be followed long-term. This follow up may be performed as part of post-licensure phase 4 studies agreed upon by the manufacturer during the licensure process. 

There is no regulatory requirement to compare the safety of the adjuvanted to the unadjuvanted vaccine formulation in comparative phase 3 safety studies. Safety information submitted to the Biologic License Application (BLA) may include the safety experience obtained from domestic or foreign trials. Safety experience with the same adjuvant formulated with other vaccine antigens may also contribute to the adjuvant’s safety evaluation.

## 4. Preclinical Studies in Animal Models

Animal models that predict safety and efficacy of a vaccine antigen, vaccine platform or adjuvant/antigen combination are often used for proof-of-concept studies prior to initiation of phase 1 trials in humans. The animal model may also assist in the initial selection of antigen dose range. However, the predictive value of animal studies for evaluation of next-generation influenza vaccines may be limited by the fact that animals are naïve, while most humans have been exposed (infected/vaccinated) to influenza since childhood and have pre-existing immune memory, which may influence their response to vaccination. Moreover, the outcome of animal challenge studies post-vaccination will vary by their susceptibility to un-adapted human and avian influenza strains, and sialic acid receptor distribution in the upper and lower respiratory tract. In addition, differential pattern recognition receptor (PRR) repertoire (targeted by various adjuvants), husbandry, microbiome, inoculation routes, immune effector functions, and the interval between vaccination and influenza virus challenge may contribute to significant differences in immune responses and protection from viral challenge compared to humans. Despite these caveats, preclinical studies may be helpful to understand vaccine induced immune responses and tease out immune mechanisms that are “reasonably likely” to predict protection. They can also be useful for identifying safety signals that can inform the design of human clinical studies to mitigate vaccination induced adverse events. An animal model that recapitulates human exposure and correlates of protection will be desirable for the preclinical evaluation of next-generation influenza vaccines.

## 5. Human Challenge Studies

FDA’s approval of Vaxchora in 2016 based on positive results from a 10 and 90-day cholera challenge trial, as well as two safety and immunogenicity trials in healthy adults that demonstrated efficacy after vaccination suggests that an appropriately designed human challenge study can provide the data to support vaccine licensure [9]. In the case of next-generation influenza vaccines, human challenge models could be useful for early evaluation and down-selection of vaccine candidate, since these studies are conducted in a defined environmental and clinical conditions [10]. A successful influenza challenge study in human subjects that shows high attack rate of confirmed infection with measurable influenza symptoms that is significantly reduced by vaccination may demonstrate “proof of concept”, supporting further development of a given vaccine platform. The model may also be appropriate for early down selection of the promising next-generation vaccines. However, there are several limitations to the current human influenza challenge studies performed in adults, as they do not replicate the influenza exposure of subjects participating in vaccine clinical trials. Firstly, adults participating in the challenge study are screened to exclude those that have already been exposed to this virus through infection or vaccination and have pre-existing immunity to the challenge virus stain. Secondly, the influenza virus strains used in the human challenge studies are typically a well characterized influenza virus strain (a purified virus with sensitivity to current influenza drugs) that circulated previously in the human population. This limitation may be addressed by developing a panel of validated challenge virus strains derived from newly circulating strains that are antigenically different from previous vaccine strains and may resemble drifted virus strains. Thirdly, most current influenza challenge strains are attenuated when compared with the circulating strains in that they do not cause severe symptoms or influenza disease. 

The utility of human challenge studies for the evaluation of next-generation influenza vaccines will need careful evaluation of viral challenge dose, routes of administration, screening assays for pre-existing immunity of subjects, study age groups, and the time interval between vaccination and viral challenge, among other factors. Availability of panel of influenza virus challenge strains that differ from the vaccine strain will help to demonstrate breadth of protection. Moreover, the careful collection of appropriate pre-and post-challenge specimens at regular time intervals that mimics natural infection can help development and evaluation of relevant immunological assays, and possibly identify correlates of protection for next-generation influenza vaccine platforms, that can be used during subsequent pivotal clinical studies.

## 6. Immunological Assays for Protective Vaccine Induced Immune Responses

Licensure of influenza vaccines in the US is based on clinical efficacy endpoints accrued during clinical trials. The production of neutralizing antibodies is a correlate of protection for many human vaccines, including currently US licensed inactivated influenza vaccines. However, no influenza vaccine has been licensed based on any immunological measurement alone. The inactivated influenza vaccines (IIVs) contain virus hemagglutinin as the active component. These vaccines are expected to induce antibodies that block interaction of circulating viruses with the host cell receptor (sialic acid) as measured in a red blood cell-based hemagglutination-inhibition (HAI) assay used as the primary in vitro immunological endpoint. HAI titers were historically shown to predict in vivo protection against influenza symptoms [11]. Most of the next-generation influenza vaccine concepts are not based on antibodies targeting the HA receptor binding domain [5,6]. Therefore, there is a need to develop new relevant immunological assays to understand and evaluate the immune mechanism of protection and ideally to identify immune surrogates “reasonably likely” to predict vaccine efficacy. These assays and correlates will be vaccine platform specific. Even though these assays or correlates of protection are not required for traditional approval of an effective vaccine, stepwise standardization/validation of the new assays between phase 1 and phase 3 will help to evaluate the immunogenicity of vaccines and guide vaccine development. Hopefully, one or more of the new immunological endpoints may be identified that correlates with clinical outcome during the pivotal phase 3 efficacy trials. Establishment of immune surrogates elicited post-vaccination that can reasonably predict protection in the clinical endpoint efficacy study could be used for post-licensure bridging clinical studies in different populations, age groups, and demographic areas, and greatly facilitates the development of future influenza vaccines.

An immune correlate of protection may also be suggested from other sources, including post-infection immunity. Even though influenza is a respiratory infection, there is paucity of relevant mucosal immune assays. Therefore, it may be prudent to develop and evaluate assays and methods to quantify mucosal immune responses in addition to measurement of systemic immunity.

## 7. Vaccine Safety

Safety of a vaccine is paramount in the licensure of any vaccine by the US FDA. As indicated above, many next-generation vaccine platforms are not designed to block virus binding to its receptor. Rather, they are likely to induce antibodies that inhibit virus replication at a post-entry stage (e.g., blocking of conformational changes/fusion in the endosomes, binding to infected cells, antibody dependent cellular cytotoxicity, antibody dependent complement activation, T cell cytotoxicity, neuraminidase inhibition) and therefore may have the potential to mediate not only virus neutralization but also enhancement of viral infection/disease [12,13,14,15,16,17,18,19,20,21,22]. Some of these alternative mechanisms, especially non-neutralizing binding antibodies and Fc-dependent activities could act as a double-edged sword that may play a detrimental role and lead to enhanced infection or enhanced disease severity [12,13,14,19,20,21,22,23]. The potential for vaccine associated enhancement of respiratory disease (VAERD) after influenza infection of vaccine recipients is a concern and vaccine sponsors should try to understand and address this possibility in various animal models, including study of lung pathology and cytokine profiling [23]. Potential product-specific safety issues may arise, and vaccine sponsors should outline their risk mitigation plans including appropriate preclinical animal models, careful clinical study design, and safety evaluation of vaccine recipients over a sufficient period after vaccination to capture exposure to influenza infections during phase 1–3 trials.

## 8. Regulatory Pathways

New vaccines must meet regulatory requirements for safety and efficacy and must be “applicable to the prevention, treatment or cure of diseases or injuries of man” (21 CFR 610.3). Only those vaccines that are demonstrated to be safe and effective, and that can be manufactured in a consistent manner will be licensed by the FDA [24]. The pivotal vaccine design should take into consideration the claims to be included in the vaccine product label. This will be particularly challenging for a multi-season influenza vaccine.

Vaccine licensure follows either a traditional approval pathway or an accelerated approval pathway. Both pathways require demonstration of product safety and consistency of vaccine manufacturing. However, traditional approval pathway includes pre-licensure evidence of efficacy from clinical trials in which influenza illness is assessed as the primary endpoint. The two licensed influenza vaccines against avian type A H5N1 strains were approved through the traditional pathway, since they used the same manufacturing process as previously licensed seasonal influenza vaccine, and they met the CBER requirements for % seroconversion (i.e., four-fold increase in HAI titers post-vaccination versus pre-vaccination) and % seroprotection (i.e., % individuals with HAI titers ≥ 1:40) For pandemic influenza vaccines wherein clinical efficacy trial cannot be performed, licensure might be pursued through accelerated approval pathway. In these instances, a surrogate immune correlate of protection is used for vaccine licensure, with a commitment to perform a clinical efficacy study in the event of an influenza pandemic.

The licensure for next-generation influenza vaccines will follow the same general pathway as for other influenza vaccines and requires a full quality package to demonstrate consistent manufacturing process in producing a safe and potent vaccine. The product-specific challenges may involve developing assays to measure vaccine potency and stability as well as the determination of an appropriate vaccine formulation, dosage, and schedule of vaccination. A non-clinical package to demonstrate the proof of concept (better, broader, “universal”) will be beneficial but not required for initiation of phase 1 trials.

Pre-marketing (pre-licensure) vaccine clinical trials are typically done in three phases: Phase 1; safety and immunogenicity studies performed in a small number of closely monitored subjects, Phase 2; dose-ranging studies that may enroll hundreds of subjects, Phase 3; may enroll thousands of individuals and provide the critical documentation of effectiveness against confirmed influenza infections and influenza-like illness (ILI). In addition to efficacy information, the Biologic License Application (BLA) should contain significant safety information collected during the pivotal phase 3 trials as well as other clinical trials with the same vaccine product. The phase 2 and phase 3 clinical trials are expected to be randomized, blinded, and include a strong statistical plan. Discussion with the FDA prior to and throughout the human clinical trials is highly encouraged.

For a broadly protective influenza vaccine, it is important to pre-define the breadth and longevity of protection and generate the data to support these claims. The case definition for influenza illness should be prospectively defined. Placebo-controlled efficacy studies in the US may be challenging given that licensed influenza vaccines are available and recommended for yearly vaccination of all age groups. Alternatively, a population at increased risk for influenza illness complications may be studied, to demonstrate “non-inferiority” or “superiority” of the new vaccine compared with a US licensed vaccine product over more than one season. However, there is no requirement to study the superiority of the vaccine unless the sponsor wants to claim “superiority” for their product.

For vaccine effectiveness studies, surveillance systems may be needed for continuous functional surveillance depending on the proposed claims of breadth and longevity of protection against influenza. This will also require identifying drift variants escaping from the immune response induced by the next-generation vaccine. It is possible that next-generation vaccines will include multiple components required to induce immunity against diverse Influenza A (Group 1 and Group 2) and Influenza B viruses. Detailed chemistry, manufacturing and control (CMC) information with validated assays to ensure manufacturing consistency and lot release criteria should be developed prior to the pivotal phase 3 trials. One or more relevant immunological endpoint assays should be identified and validated prior to initiation of the pivotal studies. It will be important to discriminate between vaccine induced responses vs. pre-existing effector functions of antibody and T cells. It is possible that repeat vaccination with the new vaccine product will be needed to maintain long term protection. Vaccine boosts may be identical or different to the primary vaccine construct. If the vaccine boost is different from the primary vaccine, then this new vaccine licensure may generate additional challenges, as currently there is no FDA licensed vaccine that includes prime-boost with different vaccine antigens. Pivotal efficacy trials may require comparison with the US standard of care against a licensed product (Quadrivalent Influenza Vaccine, Trivalent Influenza Vaccine) with follow up across several seasons, depending on the claim in the licensure package (either “non-inferiority” or “superiority” against “matched and mismatched” influenza strains). As these are new vaccine concepts, they will likely be subjected to age de-escalation criteria to perform these studies first in healthy adults followed by other age and population groups.

Regulatory pathways supporting development and approval of vaccines formulated with a novel adjuvant are the same as for unadjuvanted vaccines. Efficient planning of the development pathway for any adjuvanted vaccine requires careful attention to preclinical testing, study design, dosing decisions, and safety monitoring. Although manufacturers are not required to demonstrate the “added benefit” of adjuvanted vs. unadjuvanted vaccines in clinical comparative phase 3 studies, manufacturers should provide a justification for including an adjuvant in the vaccine. Evaluation of safety of an adjuvanted vaccine needs to include special safety considerations.

## 9. Conclusions

In summary, next-generation influenza vaccines pose significant unique challenges in development and regulation of these influenza vaccines. Some of them include development of appropriate vaccine potency assays, validated immunological assays, identifying immune correlates of protection for different vaccine platforms, development of predictive preclinical animal model for safety and efficacy and/or human challenge studies, vaccination induced long-lasting cross-protective immune responses, and definition and evaluation of what constitute improved breadth of protection during human clinical efficacy trials. Despite these challenges to make better influenza vaccines, there has been a lot of progress made over the last few years, with several new vaccine platforms already in early clinical trials. In recent years, the successful licensure of different types of influenza vaccines including cell-derived vaccine, recombinant protein vaccine and adjuvanted influenza vaccines has provided important experience that could be harnessed in the regulatory evaluation of novel influenza vaccine platforms and next-generation vaccines. Still the approval of next-generation influenza vaccines will be challenging not only for regulatory agencies and vaccine manufacturers but also for the whole influenza community. This will require dialogue, discussion, collaboration and partnership between academic/non-academic institutions, funding agencies, vaccine manufacturers, public health officials and regulatory agencies around the globe for better influenza vaccines of the world.

## Figures and Tables

**Table 1:** Quadrivalent Influenza Vaccine (QIV) licensed in the United States.

Vaccine	Manufacturer	Cell Substrate	Vaccine Dose	Vaccination Route	Indication
Fluzone	Sanofi	Egg	15 mcg HA of each vaccine strain	Intramuscular	6 months of age and above
FluzoneID	Sanofi	Egg	9 mcg HA of each vaccine strain	Intradermal	18 years through 64 years of age
Fluarix	GlaxoSmithKline	Egg	15 mcg HA of each vaccine strain	Intramuscular	6 months of age and above
FluLaval	ID Biomedical Corporation/GlaxoSmithKline	Egg	15 mcg HA of each vaccine strain	Intramuscular	6 months of age and above
FluMist	MedImmune	Egg	6.5–7.5 log_10_ FFU of live attenuated influenza virus of each vaccine strain	Intranasal	2 through 49 years of age
Flucelvax	Seqirus	MDCK cells	15 mcg HA of each vaccine strain	Intramuscular	4 years of age and above
Afluria	Seqirus	Egg	15 mcg HA of each vaccine strain	Intramuscular	5 years of age and above
Flublok	Protein Sciences	Sf9 insect cells	45 mcg HA of each vaccine strain	Intramuscular	18 years of age and above

**Table 2:** Trivalent Influenza Vaccines (TIV) licensed in the United States.

Vaccine	Manufacturer	Cell Substrate	Vaccine Dose	Vaccination Route	Indication
Fluzone HD	Sanofi	Egg	60 mcg HA of each vaccine strain	Intramuscular	65 years of age and above
Fluvirin	Seqirus	Egg	15 mcg HA of each vaccine strain	Intranasal	4 years of age and above
FLUAD	Seqirus	Egg	MF59C.1 adjuvanted; 15 mcg HA of each vaccine strain	Intramuscular	65 years of age and above
Afluria	Seqirus	Egg	15 mcg HA of each vaccine strain	Intramuscular	5 years of age and above
Flublok (Discontinued)	Protein Sciences	Sf9 insect cells	45 mcg HA of each vaccine strain	Intramuscular	18 years of age and above
